# Knowledge, Attitudes, and Practices Regarding the Disposal of Unused and Expired Medicines in Romania During the Early Implementation of the 2023 Hospital-Based Collection Framework

**DOI:** 10.3390/pharmacy14020061

**Published:** 2026-04-16

**Authors:** Anca Lupu, Ștefan Roșca, Ancuța Iacob, Marius Moroianu, Ramona-Oana Roșca

**Affiliations:** 1 Research Centre in the Medical-Pharmaceutical Field, Faculty of Medicine and Pharmacy, “Dunărea de Jos” University of Galați, 35 Al. I. Cuza Street, 800010 Galați, Romania; ancuta.dinu@ugal.ro (A.I.); moroianu.g.marius@gmail.com (M.M.); ramona.rosca@ugal.ro (R.-O.R.); 2 Medical Assistance Service of Galați Municipality, Traian Street 97, 800112 Galați, Romania; 3 Emergency Ambulance Service Galați, 1 Focșani Street, 800509 Galați, Romania

**Keywords:** medicine take-back, pharmaceutical waste, unused medicines, expired medicines, KAP survey, hospital-based collection, Romania

## Abstract

**Background:** Improper disposal of unused and expired medicines represents an environmental and public health concern. In Romania, Law No. 269/2023 assigned the responsibility for collecting household pharmaceutical waste to public and private hospitals, while operational procedures were further detailed in the Ministry of Health (MoH) Instruction No. 6226/2024. **Objectives:** This study aimed to assess knowledge, attitudes, and practices (KAP) related to the disposal of unused and expired medicines among the general public and community pharmacy staff during the early phase of implementation of the hospital-based medicine take-back system in Romania. **Methods:** A cross-sectional survey using convenience sampling was conducted between 1 and 31 August 2023. Two structured questionnaires were administered: one targeting the general public/patients and another addressing community pharmacy staff. Data were analyzed descriptively using frequencies and percentages. Several items allowed multiple responses. **Results:** Among public respondents (*n* = 108; predominantly male, 90.7%; urban, 75.0%), household waste disposal was the most frequently reported method (58.3%), followed by pharmacy return (43.5%). Willingness to use a dedicated collection system was very high (96.3%). Among pharmacy staff (*n* = 71; predominantly female, 78.9%; urban, 74.6%), 40.8% reported no collection activity; where collection occurred, it was typically on demand. Disposal routes included transfer to specialized waste companies (56.3%) and regulated destruction (43.7%). Only 1.4% of pharmacies offered incentives, while 45.4% of the public indicated discounts could motivate returns. **Conclusions:** Findings indicate an implementation and communication gap during the transition to a hospital-based pharmaceutical waste collection system. Strengthening public communication on official collection points and providing clearer operational guidance may support safer disposal practices.

## 1. Introduction

### 1.1. Pharmaceutical Waste as a Public Health and Environmental Issue

Medicines are essential to modern healthcare systems; however, their life cycle also generates a specific waste stream with implications for public health, environmental protection, and governance. Household pharmaceutical waste generally refers to unused or expired medicines stored in homes and eventually discarded, whereas pharmaceutical residues denote active pharmaceutical ingredients and metabolites that enter the environment during routine use, primarily through excretion or inappropriate disposal and releases along the pharmaceutical supply chain. Both categories contribute to the broader phenomenon known as pharmaceuticals in the environment (PiE), increasingly recognized as an emerging policy domain at the intersection of environmental protection and medication safety [1,2,3,4].

Several structural trends have amplified the relevance of pharmaceutical waste. Medicine consumption has increased due to population ageing, multimorbidity, and expanded access to pharmacological therapies. As a consequence, both pharmaceutical residues released through routine excretion and leftover medicines resulting from non-adherence, treatment modification, adverse effects, or oversupply have become more common [1,2]. At the same time, most conventional wastewater treatment plants were not designed to remove a wide spectrum of bioactive micropollutants, many of which persist at low concentrations and often occur as complex mixtures in aquatic systems [1,2,4]. Furthermore, take-back systems and safe disposal mechanisms remain unevenly implemented across countries and healthcare settings. In many cases, public awareness and engagement lag behind the regulatory frameworks intended to facilitate safe disposal, creating an implementation and communication gap that sustains inappropriate disposal routes such as household waste, sinks, or toilets [1,2,5,6,7].

Pharmaceutical substances may reach environmental compartments through multiple pathways. These include excretion during therapeutic use, household disposal of unused or expired medicines into municipal waste or wastewater systems, discharges from healthcare facilities, emissions from pharmaceutical manufacturing, and veterinary or agricultural uses, including manure spreading and aquaculture [1,2,3,8,9]. Such pathways are relevant not only for environmental protection but also for public health because bioactive compounds may persist, undergo chemical transformation, or interact additively when present in environmental mixtures [1,2,7,8,10,11,12,13].

Environmental monitoring studies have demonstrated that pharmaceutical contamination is globally widespread. Large-scale surveys of river systems have detected pharmaceutical compounds across diverse regions, with higher concentrations often associated with infrastructure constraints and substantial wastewater discharge loads [14]. Estuarine environments receiving upstream loads frequently contain pharmaceuticals and personal care products, with their fate and transport influenced by salinity gradients, sediment interactions, and hydrodynamic processes [15]. Pharmaceutical compounds and their metabolites have also been identified in sewage sludge and agricultural soils, particularly where sludge is applied as fertilizer, extending exposure pathways beyond aquatic ecosystems [16].

Ecotoxicological evidence suggests that pharmaceutical residues may affect non-target organisms even at relatively low environmental concentrations. Many pharmaceuticals are designed to act at low doses and target biological pathways that are conserved across species. Reported effects include endocrine disruption, reproductive alterations, impaired growth and development, and behavioral changes affecting feeding patterns, predator avoidance, and social interactions [14,17,18]. Under certain conditions, these effects may scale to population or ecosystem levels. A widely cited example involves the long-term reproductive disruption observed in fish populations exposed to environmental estrogens in whole-ecosystem studies, illustrating that low-dose exposure does not necessarily imply negligible ecological risk [19]. In parallel, environmental toxicology research continues to refine risk assessment frameworks for widely used pharmaceutical classes such as non-steroidal anti-inflammatory drugs [20].

In addition to environmental concerns, pharmaceutical residues have implications for public health within a One Health perspective (a concept integrating human, animal, and environmental health) [21]. Environmental exposure to antibiotic residues may contribute to selective pressure in microbial communities, potentially facilitating the emergence and spread of antimicrobial resistance (AMR), particularly in areas affected by high-load discharges from healthcare facilities or pharmaceutical manufacturing [8,22,23]. Given the global burden of AMR, reducing avoidable environmental antibiotic inputs is increasingly considered a complementary upstream measure alongside antimicrobial stewardship and infection control strategies. Moreover, unused medicines stored in households may increase the risk of accidental poisoning, medication errors, and non-medical use or diversion, particularly in households with children. These safety considerations are frequently cited as additional arguments for improving medicine take-back systems and public guidance on safe disposal [1,2,24].

Mitigation strategies combine technological, organizational, and behavioral approaches. Advanced wastewater treatment technologies, including ozonation, activated carbon filtration, and advanced oxidation processes, can reduce concentrations of certain pharmaceutical micropollutants, although effectiveness varies by compound class and operational conditions, and transformation products may pose additional uncertainties [10,24,25,26]. Consequently, many policy strategies emphasize a combination of upstream prevention measures, structured take-back systems for unused medicines, and targeted improvements in wastewater treatment where risk assessments justify such investments [1,2,3,4,10,11,12].

At the European level, pharmaceutical residues in the environment have become an important policy priority. The European Commission has proposed a strategic approach addressing PiE, emphasizing coordinated action across the entire life cycle of medicines, improved monitoring and knowledge generation, strengthened environmental risk assessment, improved disposal and take-back systems, and active engagement of stakeholders, including healthcare professionals and the public [3,20]. Regulatory agencies have also updated environmental risk assessment requirements for medicinal products, such as revisions to European Medicines Agency (EMA) guidance documents, to better integrate environmental considerations into pharmaceutical regulation [27]. Professional organizations likewise highlight the role of pharmacists and healthcare systems in promoting responsible pharmaceutical waste management and communicating appropriate disposal practices to patients [28].

Within this policy landscape, research examining knowledge, attitudes, and practices (KAP) provides valuable insights. Systematic reviews indicate that although many individuals recognize that improper disposal of medicines is undesirable, convenient but inappropriate disposal methods remain common due to limited awareness of available take-back options or uncertainty regarding the correct procedure [5,6]. Healthcare professionals and pharmacy students may also report gaps in formal training and inconsistent counseling practices related to medicine disposal, which can limit their role as facilitators of implementation [5,7]. Reviews of medicine take-back programs further highlight substantial variation in accessibility, design, and performance across countries, demonstrating that the existence of policy frameworks does not necessarily guarantee effective implementation or public utilization [29].

Taken together, pharmaceutical waste represents a complex issue at the intersection of public health, environmental protection, and healthcare governance. Its drivers include clinical prescribing patterns and patient behavior; its impacts extend across ecosystems and health security concerns such as AMR, and its solutions require coordinated infrastructure as well as credible and locally adapted public communication. These considerations justify the use of cross-sectional KAP assessments among both the general public and healthcare professionals to identify behavioral patterns and potential implementation gaps within specific health systems [1,2,3,4,5,6,7,29].

### 1.2. The Romanian Context: Regulatory Change in the Collection of Unused and Expired Medicines (Law 269/2023; MoH Instruction 6226/2024)

In 2023–2024, Romania introduced a formal regulatory framework governing the collection of unused and expired medicines originating from the population. The reform was enacted through Law no. 269/2023, which amended the national health legislation and established that unused and expired medicines from households are to be collected at designated public and private hospitals [30]. This legislative change clarified institutional responsibility and aimed to create a structured pathway for pharmaceutical waste generated at the household level.

Law no. 269/2023 does not designate community pharmacies as the official collection points for household pharmaceutical waste. Instead, it assigns responsibility to hospitals, thereby modifying previous informal or heterogeneous practices reported in different localities [30]. The law provides the legal basis for organizing the collection, temporary storage, and subsequent transfer of pharmaceutical waste to authorized operators in accordance with national waste management legislation. The reform is situated within the broader European policy context addressing PiE and safe waste management [3].

To operationalize the legislative provisions introduced by Law no. 269/2023, the Romanian Ministry of Health (MoH) issued Instruction no. 6226/2024, which provides the procedural framework for implementation at the hospital level [31]. The Instruction regulates the designation of collection points within public and private hospitals, defines the schedule under which these points must operate, and establishes the technical conditions for the temporary storage of collected medicines. It further clarifies requirements regarding the segregation of pharmaceutical waste categories, record-keeping obligations, and the transfer of collected materials to authorized waste management operators in accordance with national waste legislation. The Instruction also outlines communication responsibilities at the institutional level.

From a regulatory standpoint, the 2023–2024 reform represents a structural shift from fragmented local practices toward a centralized, hospital-based model. However, the existence of a legal framework does not automatically ensure public awareness, accessibility, or behavioral change. European policy documents have previously emphasized that effective pharmaceutical waste management requires not only regulatory clarity but also public communication, stakeholder engagement, and clear operational guidance [3].

The present study does not assess the effectiveness of the Romanian reform, nor environmental outcomes. Data were collected in August 2023, shortly after the legal clarification introduced by Law No. 269/2023, and before the issuance of the 2024 operational Instruction. Accordingly, the results should be interpreted as a baseline snapshot of how disposal practices and perceived ‘return points’ were understood and experienced before full operationalization and public communication of the hospital-based system.

The effectiveness of pharmaceutical waste regulations depends not only on legal provisions and institutional responsibilities but also on individual behaviors. In this context, the Knowledge–Attitudes–Practices (KAP) framework provides a structured approach to understanding how regulatory changes are translated into real-world action. KAP studies explore what individuals know about a given issue, how they perceive it, and how they behave in practice. In the field of unused and expired medicines disposal, this framework is particularly relevant because compliance relies heavily on voluntary participation by the public and active engagement by healthcare professionals.

Knowledge represents awareness of appropriate disposal pathways, existing collection systems, and potential environmental or health risks associated with improper disposal. Several international studies have shown that although many individuals recognize that medicines should not be discarded in household trash or flushed into sewage systems, awareness of authorized collection points and procedures is often limited [5,6,24]. Insufficient knowledge may lead to convenience-based behaviors, even when attitudes toward environmental protection are positive.

Attitudes reflect perceived importance, perceived responsibility, and moral or environmental concern related to pharmaceutical waste. Positive attitudes toward environmental protection and public safety are commonly reported in cross-sectional surveys; however, these attitudes do not consistently translate into correct disposal practices [5,24]. This discrepancy underscores the importance of examining attitudes separately from knowledge and behavior rather than assuming linear progression from awareness to action [5,6].

Practice represents actual or self-reported disposal behaviors. Systematic reviews indicate that inappropriate disposal routes, such as discarding medicines in household waste or sinks, remain common across diverse settings, including countries with established take-back programs [5,29]. Similarly, healthcare professionals and pharmacy staff may demonstrate variable counseling practices and inconsistent engagement in public education regarding disposal systems [5]. These findings suggest that regulatory availability alone does not guarantee utilization.

From an implementation perspective, KAP data serves at least three functions. First, they allow identification of information gaps that may require targeted communication strategies. Second, they help detect misalignment between public expectations and institutional design, such as when individuals report returning medicines to settings that are not officially designated collection points. Third, they provide baseline measurements in the early phase of regulatory change, enabling future monitoring of adaptation and behavioral trends without inferring causality.

European policy documents addressing PiE emphasize stakeholder engagement and public communication as critical components of successful implementation. Accordingly, assessing KAP in both the general public and frontline healthcare providers can generate implementation-relevant evidence, particularly in settings where regulatory responsibilities have recently been redefined. In the Romanian context, where hospital-based collection was formally established through Law no. 269/2023 and operationalized through MoH Instruction no. 6226/2024, KAP assessment provides a descriptive evaluation of how the new framework is perceived and understood during its early phase.

Importantly, KAP studies are observational and descriptive by design. They do not measure environmental outcomes nor establish causal relationships between regulation and behavior. Instead, they offer insight into the behavioral interface through which policy operates. In this sense, KAP functions as an implementation-sensitive lens, helping to contextualize reported practices within the broader regulatory environment [5,7].

This study provides the first empirical evidence regarding public and pharmacy staff perspectives on medicine disposal following the Romanian take-back reform.

### 1.3. Study Objectives

#### 1.3.1. Primary Objective

The primary objective of this study was to describe knowledge, attitudes, and self-reported practices regarding the disposal of unused and expired medicines among the general public and community pharmacy staff in Romania. The analysis is based on a baseline cross-sectional survey conducted in August 2023, shortly after the legal clarification introduced by Law No. 269/2023 and before the operational implementation guidance issued in 2024.

#### 1.3.2. Secondary Objectives

For the general public (patients), the secondary objectives were to characterize reported disposal routes for unused or expired medicines, assess awareness of appropriate disposal mechanisms, evaluate the perceived importance of safe disposal for environmental protection and public safety, explore willingness to use officially designated collection systems, and identify perceived facilitators or incentives that could improve participation in safe disposal practices.

For community pharmacy staff, the secondary objectives were to describe current practices related to the receipt or handling of unused or expired medicines in community pharmacy settings, assess organizational modalities and frequency of collection where applicable, document reported downstream handling procedures, evaluate opinions regarding whether medicine take-back services should be mandatory, optional, or dependent on available resources, and assess perceived patient demand for disposal services.

A cross-group objective was to descriptively compare public expectations with pharmacy-level practices within the context of the hospital-based regulatory model currently in place. The study does not evaluate policy effectiveness, environmental outcomes, or regulatory compliance and does not infer causal relationships between legislative change and observed behaviors.

## 2. Materials and Methods

This study used a cross-sectional survey design. Data were collected in Romania between 1 and 31 August 2023.

### 2.1. Study Design and Setting

The survey was conducted during the early transition period following the legislative clarification introduced by Law No. 269/2023, which established new provisions on the collection of unused and expired medicines from the population, and before the issuance of the MoH’s operational guidance (Instruction No. 6226/2024).

The study aimed to assess knowledge, attitudes, and self-reported practices regarding the disposal of unused and expired medicines among members of the public and community pharmacy staff.

### 2.2. Participants, Sampling, and Recruitment

Two respondent groups were included in the study:

1. Members of the general public (*n* = 108);
2. Community pharmacy staff (*n* = 71).

Participants were recruited using a convenience sampling strategy. Members of the public were approached in pharmacies and via online distribution of the survey link. Pharmacy staff were recruited through professional contacts in community pharmacies.

Eligibility criteria included age ≥ 18 years and voluntary participation in the survey. No stratified sampling by sex, age, or residence was applied.

The total number of individuals approached cannot be determined retrospectively, as distribution relied on both in-person contact in pharmacy settings and online forwarding of the survey link—methods that do not permit tracking of non-responders. Consequently, a formal response rate cannot be calculated, which represents an additional limitation (see Section 4.5). No formal sample size calculation or power analysis was conducted before data collection. The study was designed as a descriptive, exploratory cross-sectional survey using convenience sampling as a pragmatic approach given the absence of a readily accessible sampling frame. Pharmacy staff were recruited through professional contact rather than through a central register or professional body. For context, according to the National Institute of Statistics (INS), Romania had approximately 21,200 pharmacists and approximately 8400 community pharmacies in 2024 (INS, Reteaua unitatilor sanitare in anul 2024, June 2025). The staff sample (*n* = 71), therefore, represents a small convenience sample, and results cannot be generalised to the national pharmacy workforce [32].

### 2.3. Survey Instruments and Variables

Two structured questionnaires were developed specifically for this study: one addressed to the general public, and one addressed to pharmacy staff.

The questionnaire for the general public assessed disposal practices for unused or expired medicines, frequency of medicine disposal, experiences with attempting to return medicines, perceived importance of safe disposal, preferred solutions for improving collection systems, and potential incentives for participation in medicine take-back initiatives.

The questionnaire for pharmacy staff included items regarding professional role, pharmacy setting, current collection practices for unused or expired medicines, collection frequency, handling of returned medicines, perceived patient demand, and attitudes toward mandatory medicine collection services.

Some items allowed multiple responses (e.g., disposal routes or reasons for safe disposal), while others were designed as single-choice questions.

The questionnaire for the general public comprised 9 items covering demographic characteristics (age group, sex, and residence) and KAP domains (disposal practices, disposal frequency, return experience, motivations for proper disposal, emotional responses, willingness to use dedicated collection systems, preferred solutions, and preferred incentives). The questionnaire for pharmacy staff comprised 14 items covering professional role, pharmacy type and setting, collection practices, collection frequency, handling of collected medicines, preferred public information measures, opinion on mandatory collection, perceived patient demand, incentive provision, and perceived effectiveness of incentives. Both instruments were developed for this study, informed by the existing KAP literature, and reviewed internally before administration. Formal external piloting was not conducted, which represents a methodological limitation. Both questionnaires are provided as Supplementary Material (Supplementary Files S1 and S2).

### 2.4. Data Management and Statistical Analysis

Survey responses were exported from Google Forms into Microsoft Excel for data cleaning and descriptive analysis.

Statistical analysis was limited to descriptive statistics. Categorical variables are presented as frequencies and percentages (*n*, %). For items allowing multiple responses, percentages were calculated using the total number of respondents as the denominator; therefore, totals may exceed 100%.

No inferential statistical testing was performed.

The decision not to perform inferential statistical testing was made a priori and reflects three constraints: (i) convenience sampling does not support population-level inference; (ii) individual-level raw data were unavailable, as aggregated response counts were extracted from Google Forms, precluding multivariable modelling; and (iii) several multi-response items do not satisfy assumptions required for standard inferential tests. To provide additional context, 95% confidence intervals (Wilson score method) are reported alongside primary outcome proportions in Section 3.

### 2.5. Ethical Considerations

Participation in the survey was voluntary and anonymous. No personally identifiable information was collected. Completion of the questionnaire was considered to represent implied informed consent to participate in the study.

In accordance with Romanian national regulations and institutional policy, ethical review and approval were waived for this study because (i) the research involved a fully anonymous voluntary survey; (ii) no personal or sensitive data were collected; (iii) participants could not be identified at any stage; and (iv) the study did not involve clinical interventions, biological samples, or vulnerable populations. The study complied fully with the ethical principles outlined in the Declaration of Helsinki (2013 revision). All participants provided implied informed consent through voluntary completion of the questionnaire, preceded by an information statement describing the study purpose, voluntary nature of participation, and anonymity of responses.

## 3. Results

### 3.1. Participant Characteristics

A total of 179 respondents completed the survey, including 108 members of the public and 71 community pharmacy staff (Table 1).

Participant characteristics are summarized in Table 1. The public sample was predominantly male (90.7%) and urban (75.0%), with most respondents aged 31–40 years (48.1%). The pharmacy staff sample was predominantly female (78.9%) and urban (74.6%), with pharmacy assistants representing the largest staff category (53.5%); no respondents worked in hospital pharmacies.

### 3.2. Public Survey—Practices and Attitudes

#### 3.2.1. Methods of Disposal (Multiple Responses Allowed)

As this was a multi-selected item, percentages exceed 100%. Notably, despite the current Romanian legal framework designating hospitals as the official collection points, a substantial proportion of respondents reported returning medicines to pharmacies, suggesting a potential implementation or communication gap between policy and public understanding. Detailed distributions of public practices and attitudes are presented in Table 2.

#### 3.2.2. Frequency of Disposal and Return Experiences

Regarding the frequency of medicine disposal, most respondents reported disposal of medicines rarely (57/108; 52.8%). A further 23/108 (21.3%) stated they had never disposed of medicines, while 19/108 (17.6%) did so often, and 9/108 (8.3%) very often.

When asked about previous attempts to return unused or expired medicines:

- 33/108 (30.6%) reported that they had attempted to return medicines and that these were accepted;
- 23/108 (21.3%) had attempted to return them but were refused;
- 52/108 (48.1%) had never attempted to return medicines.

These findings indicate variability in practice across pharmacy settings. As community pharmacies have no statutory obligation to collect household pharmaceutical waste under Law No. 269/2023, the proportion reporting no collection activity is consistent with the current legal framework.

**Figure 1:**
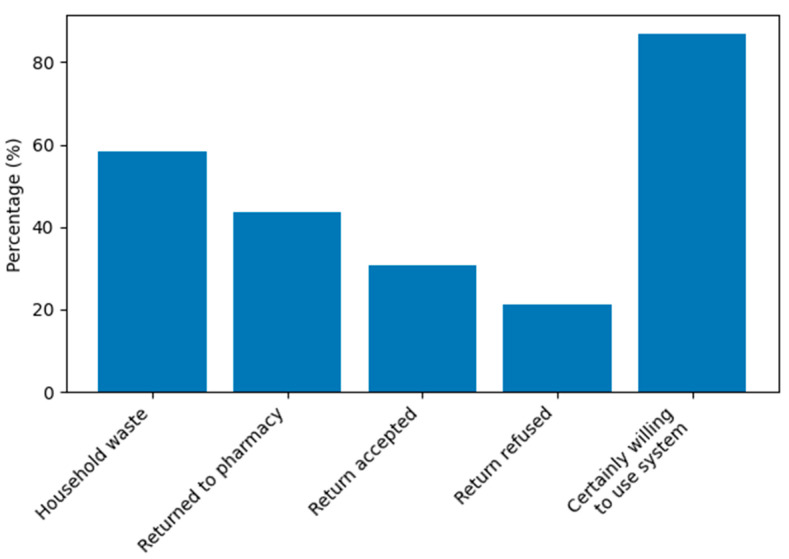
Public practices versus willingness to use structured collection systems.

As shown in Figure 1, household disposal remains common despite a very high declared willingness to use dedicated medicine take-back systems.

#### 3.2.3. Motivation, Emotions, and Willingness to Use Dedicated Systems

Environmental protection was the most frequently endorsed motivation for proper disposal (77.8%), followed by prevention of unauthorized access (66.7%) and accidental poisoning (53.7%); only 1.9% considered proper disposal unimportant. Emotional responses to improper disposal were predominantly negative (57.4% guilty, 37.0% worried), and willingness to use dedicated collection systems was very high (96.3% certainly or probably). Full distributions are provided in Table 2.

Overall, the findings suggest a discrepancy between current disposal practices, where household waste remains common, and strong attitudinal support for structured, accessible, and clearly communicated collection systems.

### 3.3. Pharmacy Staff Survey—Practices and Attitudes

#### 3.3.1. Collection Practices

Collection practices varied substantially across pharmacy settings (Table 3). Notably, 40.8% of staff reported no collection activity, while 39.4% reported collecting both unused and expired medicines.

#### 3.3.2. Collection Interval: Actual Versus Preferred

On-demand collection was the most frequently reported current interval (39.4%) and the most preferred option (36.6%), though 25.4% of staff expressed no preference for implementing collection services (Table 3).

Overall, while on-demand collection was the most frequently reported and preferred option, a notable proportion of staff expressed no preference for implementing collection services.

#### 3.3.3. Management of Collected Medicines

Reported downstream handling routes included transfer to a specialized waste management company (56.3%) and regulated destruction (43.7%). Totals exceed 100%, suggesting multiple responses or reporting inconsistencies; results should be interpreted cautiously (Table 3).

#### 3.3.4. Public Information Measures

Mass media campaigns were most frequently endorsed as a public information strategy (39.4%), followed by community campaigns (23.9%) and informational leaflets in pharmacies (21.1%) (Table 3).

It should be noted that these responses reflect staff perceptions collected in August 2023. Contemporary evidence suggests that digital communication strategies—including targeted social media campaigns, pharmacy chain mobile applications, and AI-assisted patient information platforms—may offer more cost-effective and wider-reach alternatives to traditional mass media, particularly among younger age groups [21]. Future implementation strategies in Romania may benefit from integrating these channels alongside traditional approaches.

#### 3.3.5. Opinion Regarding Mandatory Collection Services

Opinions on mandatory take-back were divided: 33.8% favored mandatory service, 28.2% preferred an optional approach, and 25.4% considered implementation should depend on available resources. No clear professional consensus emerged (Table 4).

Overall, staff responses indicate limited implementation of structured take-back practices, modest perceived patient demand, and divided views regarding regulatory obligation and the role of incentives.

#### 3.3.6. Illustrative “Demand–Availability” Gap Indicators (Public vs. Pharmacy Staff)

While these measures capture different constructions and were assessed in different populations, the contrast provides a quantitative signal of potential misalignment between public willingness and reported service availability. Incentives showed a similar asymmetry: only 1.4% of staff reported offering incentives (1/71; 0.2–7.6), whereas 45.4% of the public indicated that discounts could motivate returns (49/108; 36.3–54.8)

## 4. Discussion

### 4.1. Main Findings

Aligned with our objective to describe public disposal practices and attitudes, the survey indicates that suboptimal disposal remains common, with household waste the dominant route, alongside smaller but non-negligible reports of disposal via sink/toilet and burning. At the same time, support for structured take-back is very high, and respondents frequently endorsed system-level solutions (e.g., dedicated kiosks/collection points) and non-monetary motivations tied to environmental and public health protection. This pattern, positive attitudes coexisting with inadequate practices, is consistent with recent KAP evidence syntheses in household pharmaceutical waste management [5,33].

In relation to the objective of assessing experiences with returning medicines, half of the public had attempted to return medicines, and among those who tried, refusal was common, suggesting inconsistent service access at the point of return. Given that data were collected in August 2023, shortly after the legal clarification introduced by Law 269/2023 and before the 2024 operational guidance (Instruction 6226/2024), these findings reflect an early transition period. This mismatch supports our objective of identifying a communication gap during the early transition to a hospital-based system [34,35].

Regarding the objective of characterizing pharmacy staff practices and implementation capacity, staff responses suggested heterogeneity: a substantial share reported no collection activity, while others reported collecting expired and/or unused medicines, typically on an on-demand basis. Staff also reported downstream handling routes centered on transfer to specialized waste companies and emphasized mass media/community campaigns as preferred public information strategies—findings that align with broader literature noting that availability, clarity of guidance, and accessibility of take-back options are recurring barriers even when attitudes are favorable [36,37]. Taken together, these results sit within the wider European policy context that requires Member States to ensure appropriate collection systems for unused/expired medicines and reflects ongoing EU-level attention to pharmaceutical residues in the environment [38,39].

### 4.2. Interpretation in Policy Context: An Implementation/Communication Gap

Romania’s current framework for household pharmaceutical waste has been explicitly clarified in recent legislation. Law 269/2023 amended the health reform law to state that expired and/or unused medicines from the population are collected by public or private hospitals, which are obliged to receive them for final disposal, with financing mechanisms also specified [30]. To operationalize this requirement, MoH Instruction 6226/2024 sets procedural rules for managing these medicines as waste streams originating from the population [31]. In parallel, the national medicines authority (ANMDMR) disseminated implementation-oriented guidance indicating that patient-facing materials should include a prominent warning that expired/unused medicines must be returned to public or private hospitals, reinforcing the intended collection point in routine risk communication [40].

Within this policy setting, our findings are most plausibly interpreted as a misalignment between “policy-as-written” and “policy-as-understood/experienced”. Public respondents frequently reported disposal via household waste and, notably, many reported returning medicines to pharmacies—despite the hospital-based collection requirement—while pharmacy staff reported heterogeneous practices, including a substantial proportion reporting no collection activity. Rather than implying any causal effect of legal changes, these patterns are consistent with an implementation/communication gap, where citizens and front-line actors do not converge on a single, clearly understood, and consistently available disposal route. This interpretation mirrors broader KAP evidence that positive attitudes toward safe disposal often coexist with persistent improper practices when collection pathways are unclear or inconvenient [40].

At the EU level, Member States are expected to ensure that appropriate collection systems exist for unused/expired medicines, but the Directive is not prescriptive about the exact operational model [39]. The EU’s Strategic Approach to PiE explicitly frames improper disposal as one contributor to environmental contamination and highlights the role of awareness-raising and waste reduction measures across the medicine lifecycle [3]. In this sense, Romania’s hospital-centered approach can be viewed as one compliance pathway, but its real-world effectiveness depends on visibility, accessibility, and consistent messaging across healthcare touchpoints (hospitals, pharmacies, media, and patient information).

International policy and technical guidance support this emphasis on communication and system design. The OECD highlights that well-targeted communication campaigns, including clear disposal instructions on packaging/leaflets, are important to improve awareness and reduce improper routes such as flushing or discarding in household waste [1]. Likewise, World Health Organization (WHO) technical guidance on pharmaceutical disposal underscores the need for organized systems to prevent environmental release and unsafe handling, reinforcing the rationale for structured collection routes [41]. Taken together, the policy implication is not that the Romanian framework is ineffective, but that the translation from regulation to routine public behavior may require: (i) sustained, multi-channel communication beyond formal legal publication; (ii) consistent point-of-care counseling and signage across hospitals and community pharmacies; and (iii) practical, user-centered access arrangements (e.g., predictable schedules, clear locations, and standardized procedures). These actions align with EU and OECD emphases on awareness and system accessibility as enabling conditions for safe disposal behaviors [1].

### 4.3. Comparison with Literature

Across Europe and other settings, surveys consistently show a “KAP paradox” in which environmental and safety concerns are high, yet household disposal (bin/trash) and occasional flushing remain frequent. In a recent systematic review of cross-sectional studies on unused-medicine disposal, the average return rate to formal take-back facilities was low overall, although European studies reported higher return rates than other regions, with notable variation by country [42]. This broader pattern is consistent with our public sample, where household waste was the most commonly reported route, while willingness to use a structured system was very high.

#### 4.3.1. Public Practices: “Trash Still Dominates,” with Wide Cross-Country Variability

Our estimate for disposal of household waste is within the range reported in European household surveys and case studies, where bin/trash often remains the predominant route. For example, a Polish case study reported that many respondents disposed of pharmaceuticals in household waste (and some by flushing), while a minority returned medicines to pharmacies [43]. A UK survey similarly found that disposal behaviors largely clustered into bin, sink/toilet, and pharmacy channels, and highlighted that awareness and receipt of information about pharmacy return can be incomplete [44]. These findings mirror our interpretation that attitudes alone are insufficient when information and access pathways are not clear or consistently reinforced.

#### 4.3.2. Return Behavior: Higher “Return to Pharmacy” Proportions Resemble High-Performing European Contexts, but Measures Are Not Directly Comparable

In our survey, many public respondents reported returning medicines to pharmacies (multi-select item), a proportion that sits at the higher end of reported European return behavior. In Sweden, repeated population surveys reported high knowledge of pharmacy return and substantial actual return within the preceding year [45]. Cross-country syntheses also show that some European countries (e.g., Sweden, Ireland, UK) have return rates higher than the global average, though still far from universal [42]. Importantly, cross-study comparability is limited by differences in question wording (e.g., “ever returned,” “returned in last 12 months,” or “usual method,” and whether multiple responses are allowed), sampling frames, and the maturity of national schemes.

#### 4.3.3. High Willingness Does Not Necessarily Translate into Uptake

The literature suggests that willingness and actual return behavior are only weakly correlated in many settings, reinforcing the idea that systems need to be easy to use and widely communicated [42]. Our results fit this pattern: stated willingness to use dedicated systems was very high, yet reported household disposal remained common—an observation consistent with policy analyses emphasizing that convenience, visibility, and consistent guidance influence real-world disposal more than attitudes alone [1,3].

#### 4.3.4. Mature EU Schemes Show What High Uptake Can Look Like

In countries with long-standing, pharmacy-based collection models and intensive communication, reported uptake can be higher. France’s Cyclamed reporting indicates that a large majority of citizens say they return unused/expired medicines to pharmacies, and environmental protection is frequently cited as a motivation [46,47]. Spain’s SIGRE system similarly reports extensive nationwide infrastructure and year-on-year increases in collection via pharmacies, suggesting that stable logistics plus sustained public communication can normalize the return behavior [48]. At the EU policy level, Directive 2001/83/EC requires Member States to ensure appropriate collection systems for unused/expired medicines, while leaving operational details to national implementations supporting the plausibility of varied national models (pharmacy- vs. hospital-centered) with different communication demands [39].

The factors commonly associated with high uptake in these mature schemes include: (i) pharmacy-centered infrastructure with high geographic density and predictable opening hours; (ii) sustained multi-channel public communication maintained over years, creating behavioral norms around medicine return; (iii) clear professional role definition, with pharmacists explicitly trained and mandated to accept returned medicines; (iv) transparent logistics with publicly reported collection volumes, reinforcing public confidence; and (v) extended producer responsibility frameworks providing financial sustainability. Romania’s hospital-centered model may require adaptation of these principles to its specific institutional context [4,6,49].

### 4.4. Pharmacy Staff Practices: Our Heterogeneity Aligns with Evidence of Training/Role-Clarity Gaps

A recent systematic review on healthcare staff and students found that, despite positive attitudes, knowledge, and practice gaps persist, including limited awareness of available services and inconsistent application of recommended disposal routes, especially outside tightly regulated healthcare waste streams. The heterogeneity observed among community pharmacy staff, including a substantial proportion reporting no collection activity, variable preferred collection frequencies, and diverse downstream handling routes, is consistent with the broader literature documenting knowledge and practice gaps among healthcare professionals regarding pharmaceutical waste disposal [37].

#### Waste Stream Evidence Complements Survey Data

Beyond surveys, waste stream analyses from European cities show that unused medicines still appear in household garbage, underscoring that improper disposal is not just a self-report artifact. A study from Vienna documented medicines discarded in household waste, illustrating the persistence of leakage into municipal waste even where take-back options exist [50]. Similarly, analyses of medicines returned to pharmacies in Italy show that large quantities and costs are associated with returned (often expired) packages, highlighting the scale of unused medicines and the potential value of efficient collection systems [51].

In sum, our results are consistent with European evidence that (i) household disposal remains prevalent, (ii) return behavior varies widely, and (iii) high willingness/awareness does not guarantee uptake without clear, repeatedly communicated, and accessible collection pathways [42].

### 4.5. Implications

#### 4.5.1. Implications for Public Communication: Hospitals as the Official Collection Point

Under the current Romanian framework, public and private hospitals are designated as the official collection points for unused and expired medicines originating from the population, as established by Law 269/2023 and further operationalized through MoH Instruction 6226/2024. This regulatory clarification represents a structural shift from informal or pharmacy-centered expectations toward a hospital-based model.

Our findings suggest that, while public willingness to use structured collection systems is high, a substantial proportion of respondents reported either disposing of household waste or returning medicines to pharmacies. These patterns indicate that legal designation alone may be insufficient without sustained, visible, and repetitive communication strategies.

European policy documents emphasize that effective collection systems require not only legal mandates but also clear public information and practical accessibility. Directive 2001/83/EC requires Member States to ensure appropriate collection systems for unused medicinal products but does not prescribe a specific operational model, thereby placing responsibility on national authorities to ensure functional implementation and communication clarity [52]. The European Commission’s Strategic Approach to PiE further highlights awareness-raising and citizen engagement as essential components of minimizing improper disposal routes [3].

OECD analyses similarly underline that public awareness campaigns, standardized disposal instructions, and harmonized messaging across healthcare touchpoints are key determinants of behavioral uptake [1]. WHO guidance on pharmaceutical waste management also stresses the importance of organized, well-communicated systems to prevent environmental release and unsafe handling [41].

Based on the findings, the following concrete measures are proposed for the Romanian context:

(i) Hospital-level implementation: Public and private hospitals designated as collection points under MoH Instruction No. 6226/2024 should display standardized trilingual signage (Romanian, Hungarian, and Roma, where demographically relevant) indicating the location, schedule, and accepted waste categories of their collection point. Discharge documentation and patient information leaflets should include a dedicated section on medicine return, with the hospital address and collection schedule.

(ii) Cross-sector communication: The Romanian National Agency for Medicines and Medical Devices (ANMDMR) should coordinate a unified public communication campaign across hospitals, community pharmacies, and primary care providers, ensuring consistent messaging regardless of the collection point visited by the patient. Although community pharmacies are not officially designated as collection points, they remain an important perceived access point for patients.

(iii) Digital and point-of-sale communication: Given that the study population included a high proportion of urban residents with internet access, digital communication channels (ANMDMR website, health authority social media accounts, and pharmacy chain applications) should be used to publicize hospital collection schedules and locations. QR codes linking to a national map of collection points could be displayed in pharmacies and on medicine packaging.

(iv) Incentive framework: Although financial incentives are not currently provided under the Romanian framework, the finding that 45.4% of the public cited discounts as a motivating factor suggests that a voluntary pharmacy-based incentive scheme (e.g., loyalty points for verified medicine return at hospitals, redeemable at pharmacies) could increase uptake without requiring statutory change.

Such measures align with international evidence that behavioral compliance increases when disposal pathways are simple, visible, and consistently reinforced [9].

#### 4.5.2. Implications for Community Pharmacies: Perceived Role Versus Legal Role

A second implication concerns the position of community pharmacies within the current Romanian system. Although the legal framework assigns collection responsibility to hospitals, many public respondents reported returning medicines to pharmacies, and pharmacy staff responses indicated heterogeneous practices, including some ongoing collection activities.

This discrepancy reflects a potential divergence between the perceived role of pharmacies (as accessible, medication-focused healthcare providers) and their current legal role under the hospital-centered model. Internationally, pharmacy-based take-back systems are common and often well-established (e.g., Sweden, France, Spain), where pharmacies serve as the primary interface for medicine return [45,46,53]. These models have demonstrated that pharmacies can function effectively as collection hubs when supported by standardized coordination and communication frameworks.

It bears emphasis that community pharmacies in Romania have not acted contrary to any legal requirement. Under Law No. 269/2023, the statutory obligation for collecting household pharmaceutical waste rests with hospitals, not community pharmacies. Any collection activities reported by pharmacy staff in this study, therefore, represent supererogatory practice, voluntary service provision beyond legal obligation. Several arguments support voluntary pharmacy engagement: (i) pharmacies are the point of medicine dispensing and a natural locus of medicine-related public education; (ii) patient expectations, as documented in this study, where 43.5% of the public reported returning medicines to pharmacies, suggest that pharmacies are perceived as the primary return point regardless of legal designation; (iii) voluntary participation in take-back could support pharmacies’ professional image and public health role. However, voluntary engagement would require clear operational guidance, waste management infrastructure, and coordination with authorized disposal operators.

However, even in systems where pharmacies are designated collection points, the literature documents variability in staff knowledge, operational clarity, and counseling practices. A recent systematic review found persistent knowledge–practice gaps among healthcare professionals regarding appropriate disposal pathways [37]. This suggests that simply expanding or modifying pharmacy roles without parallel investment in training and guidance may not automatically resolve inconsistencies.

Importantly, these implications do not presuppose that pharmacies should or should not collect medicines but rather highlight the need for role coherence between legislation, professional expectations, and public perception. Where public expectations center on pharmacies as the natural return point, communication strategies must either clearly redirect behavior to hospitals or formally integrate pharmacies into the system to avoid confusion.

### 4.6. Implications for Future Research

The present study generates several hypotheses warranting testing in future, more robustly designed investigations. First, representative population surveys using stratified probability sampling—with adequate power for subgroup comparisons by sex, age, residence, and educational level—would enable inferential analysis of factors associated with disposal behavior. Second, prospective monitoring studies conducted after full operationalisation of MoH Instruction No. 6226/2024 would allow assessment of behavioral change relative to the baseline documented here. Third, qualitative research with community pharmacy staff and hospital-based collection point staff would provide deeper insight into perceived barriers and implementation challenges. Fourth, comparative studies across Romanian regions or with other EU Member States that have recently reformed pharmaceutical take-back frameworks could identify system-level factors associated with higher uptake.

### 4.7. Strengths and Limitations

This study has several strengths. First, it captured perspectives from two stakeholder group members of the public and community pharmacy staff, allowing triangulation of reported behaviors, perceived service availability, and attitudes relevant to implementation. Second, the survey addressed not only practices, but also motivations, emotions, and policy preferences, which are directly actionable for communication and service design. Third, the findings were generated in the context of Romania’s evolving take-back framework, providing timely descriptive evidence to inform rollout and public information strategies.

Several limitations should be considered when interpreting the results. The study used convenience sampling, which increases the likelihood of selection bias (e.g., over-representation of individuals more engaged with health services) and limits external validity/generalizability beyond the sampled settings. The public sample showed a marked sex imbalance, suggesting that respondent composition may not reflect the broader population structure and may influence the distribution of attitudes and practices. All outcomes were self-reported, which introduces potential recall bias and social desirability bias, particularly for behaviors with clear normative expectations (e.g., environmentally responsible disposal).

The absence of a calculable response rate, resulting from the combined in-person and online distribution strategy, prevents assessment of non-response bias and constitutes an additional limitation. Similarly, the questionnaires were not formally piloted before administration, which limits confidence in the consistency of item interpretation across respondents.

Because the design was cross-sectional, the results are descriptive and do not support causal inference regarding the effects of the policy framework or communication efforts. In addition, the absence of individual-level raw data prevents multivariable modeling and restricts analysis to aggregated distributions and derived indicators. Finally, a small number of items were multi-selected (and at least one staff item may reflect multi-response and/or reporting inconsistencies), which could complicate interpretation if respondents understood response options differently or if the questionnaire format was not uniform across administrations.

The study relied exclusively on descriptive analyses due to the use of aggregated survey data. No inferential statistical testing was conducted. Therefore, findings should be interpreted as exploratory and hypothesis-generating rather than confirmatory.

A marked sex imbalance was observed in the public sample, with a predominance of male respondents (90.7%). This distribution does not reflect the demographic structure of the general population and is the result of convenience sampling, including in-pharmacy recruitment and online forwarding of the survey link. Consequently, the findings may not be fully generalizable to the broader Romanian population, particularly to women, who may have different health-seeking behaviors and medicine disposal practices.

Overall, these constraints mean the study is best interpreted as a practice- and implementation-oriented snapshot that can guide targeted communication and future, more representative evaluations.

## 5. Conclusions

In this cross-sectional survey, household disposal of unused or expired medicines was common among public respondents, although willingness to use dedicated take-back systems was very high. Many respondents reported returning medicines to pharmacies, and some experienced refusal when attempting to return them, indicating inconsistent return experiences. Pharmacy staff reported heterogeneous collection practices, with a substantial proportion indicating no collection activity and mixed preferences regarding collection frequency and mandatory service provision. Together, these findings suggest a practical mismatch between reported behaviors, service availability, and the intended hospital-based collection pathway. Strengthening clear, consistent public communication on official collection points and standardizing frontline guidance may support safer disposal practices.

Future representative surveys and prospective monitoring studies, conducted following the full operationalisation of MoH Instruction No. 6226/2024, are warranted to assess the evolution of disposal behaviors and public awareness as the hospital-based collection framework becomes established.

## Figures and Tables

**Table 1:** Participant characteristics (public respondents and community pharmacy staff).

Characteristic	Public (*n* = 108), *n* (%)	Pharmacy Staff (*n* = 71), *n* (%)
Age group (years)		
18–30	25 (23.1)	19 (26.8)
31–40	52 (48.1)	31 (43.7)
40–50	25 (23.1)	14 (19.7)
>50	6 (5.6)	7 (9.9)
Sex		
Female	10 (9.3)	56 (78.9)
Male	98 (90.7)	15 (21.1)
Residence/work setting		
Urban	81 (75.0)	53 (74.6)
Rural	27 (25.0)	18 (25.4)
Staff role		
Pharmacy assistant	—	38 (53.5)
Pharmacist	—	17 (23.9)
Chief pharmacist	—	11 (15.5)
Other/unspecified	—	5 (7.1)
Pharmacy type		
Independent	—	24 (33.8)
Chain	—	47 (66.2)
Hospital pharmacy	—	0 (0.0)

Values are *n* (%). Percentages may not come to 100 due to rounding. “Other/unspecified” indicates staff roles not classified into the predefined categories. “—” = not applicable.

**Table 2:** Public survey—practices and attitudes (*n* = 108).

Variable	*n* (%)	95% CI
**Methods of disposal ᵃ**		
Household waste (trash)	63 (58.3)	48.6–67.5
Returned to the pharmacy	47 (43.5)	34.2–53.2
Sink/toilet	9 (8.3)	4.3–15.3
Burning	5 (4.6)	1.9–10.5
**Frequency of disposal**		
Very often	9 (8.3)	4.3–15.3
Often	19 (17.6)	11.4–26.1
Rarely	57 (52.8)	43.1–62.3
Never	23 (21.3)	14.5–30.1
**Return attempt experience**		
Attempted and accepted	33 (30.6)	22.4–40.1
Attempted and refused	23 (21.3)	14.5–30.1
Never attempted	52 (48.1)	38.6–57.8
**Reasons for proper disposal ᵃ**		
Protect the environment	84 (77.8)	68.9–84.8
Prevent unauthorized access/incidents	72 (66.7)	57.1–75.1
Prevent accidental poisoning	58 (53.7)	44.1–63.1
Not important	2 (1.9)	0.5–6.5
**Emotional response when discarding improperly**		
Guilty	62 (57.4)	47.7–66.6
Worried	40 (37.0)	28.2–46.8
Uneasy	6 (5.6)	2.6–11.7
Indifferent	0 (0.0)	—
**Willingness to use dedicated collection systems**		
Certainly	94 (87.0)	79.2–92.3
Probably	10 (9.3)	5.1–16.5
Unsure	2 (1.9)	0.5–6.5
Certainly not	2 (1.9)	0.5–6.5
**Preferred solutions**		
Special collection vending machines	54 (50.0)	40.6–59.4
Legal obligation for medical units	37 (34.3)	25.7–43.9
Education/training	17 (15.7)	9.9–24.0
No solutions	0 (0.0)	—
**Preferred incentives**		
Protect the environment/public health	55 (50.9)	41.5–60.3
Discounts	49 (45.4)	36.2–54.9
Prize draws	1 (0.9)	0.2–5.0
Nothing	3 (2.8)	1.0–8.0

ᵃ Multiple responses permitted; percentages are calculated using the total number of respondents (*n* = 108) as the denominator and may therefore exceed 100%. 95% CI = 95% confidence interval calculated using the Wilson score method. For items with 0 responses, a confidence interval is not applicable (—). Values are presented as *n* (%). Percentages for single-choice items may not sum to 100% due to rounding.

**Table 3:** Pharmacy staff survey—Collection practices (*n* = 71).

Variable	*n* (%)	95% CI
**Collection practice**		
Unused medicines only	2 (2.8)	0.8–9.7
Expired medicines only	12 (16.9)	9.9–27.1
Both unused and expired medicines	28 (39.4)	28.7–51.1
No collection	29 (40.8)	29.9–52.6
**Current collection interval**		
Monthly	9 (12.7)	6.7–22.6
Quarterly	8 (11.3)	5.8–20.9
On demand	28 (39.4)	28.7–51.1
No collection	26 (36.6)	26.2–48.4
**Preferred collection interval** ᵇ		
Monthly	18 (25.4)	16.5–36.8
Quarterly	9 (12.7)	6.7–22.6
On demand	26 (36.6)	26.2–48.4
No collection system	18 (25.4)	16.5–36.8
**Handling of collected medicines** ᵃ		
Transferred to a specialized waste company	40 (56.3)	44.5–67.5
Destroyed appropriately (regulated channels)	31 (43.7)	32.5–55.5
Donated to hospitals	2 (2.8)	0.8–9.7
Returned to stock	0 (0.0)	—
**Preferred public information measure**		
Mass media campaigns	28 (39.4)	28.7–51.1
Community campaigns	17 (23.9)	15.2–35.3
Informational leaflets in pharmacies	15 (21.1)	13.1–32.2
School curriculum	11 (15.5)	8.9–25.7
**Opinion on mandatory take-back service**		
Should be mandatory	24 (33.8)	23.5–45.7
Optional	20 (28.2)	18.8–39.8
Depending on resources	18 (25.4)	16.5–36.8
No opinion	9 (12.7)	6.7–22.6
**Perceived monthly patient demand**		
1–10 requests	50 (70.4)	58.8–79.9
11–50 requests	6 (8.5)	3.9–17.4
51–100 requests	0 (0.0)	—
>100 requests	0 (0.0)	—
None	15 (21.1)	13.1–32.2
**Incentives currently offered**		
Yes	1 (1.4)	0.3–7.6
No	70 (98.6)	92.4–99.7
**Perceived effectiveness of incentives**		
Yes, effective motivation	23 (32.4)	22.3–44.3
Depending on the incentive type	9 (12.7)	6.7–22.6
No awareness should be the main driver	31 (43.7)	32.5–55.5
No—risk of misuse/abuse	8 (11.3)	5.8–20.9

ᵃ Multiple responses permitted; percentages are calculated using the total number of respondents as the denominator and may therefore exceed 100%. Totals exceeding n reflect multiple responses or reporting inconsistencies; interpret cautiously. ᵇ Preferred collection interval reflects the personal preference of pharmacy staff respondents regarding the frequency they would consider most appropriate if collection were to be implemented or continued in their pharmacy; it does not refer to current practice. 95% CI = 95% confidence interval calculated using the Wilson score method. For items with 0 responses, a confidence interval is not applicable (—). Values are presented as *n* (%). Percentages for single-choice items may not sum to 100% due to rounding.

**Table 4:** Pharmacy staff survey—Opinions on mandatory take-back (*n* = 71).

Variable	*n* (%)
Collection practice	
Unused medicines only	2 (2.8)
Expired medicines only	12 (16.9)
Both unused and expired medicines	28 (39.4)
No collection	29 (40.8)
Current collection interval	
Monthly	9 (12.7)
Quarterly	8 (11.3)
On demand	28 (39.4)
No collection	26 (36.6)
Preferred collection interval	
Monthly	18 (25.4)
Quarterly	9 (12.7)
On demand	26 (36.6)
No collection	18 (25.4)
Handling of collected medicines ^†^	
Transferred to a specialized waste company	40 (56.3)
Destroyed appropriately (regulated channels)	31 (43.7)
Donated to hospitals	2 (2.8)
Returned to stock	0 (0.0)
Preferred public information measure	
Mass media	28 (39.4)
Community campaigns	17 (23.9)
Leaflets in pharmacies	15 (21.1)
School curriculum	11 (15.5)
Opinion on mandatory take-back service	
Should be mandatory	24 (33.8)
Optional	20 (28.2)
Depending on resources	18 (25.4)
No opinion	9 (12.7)
Perceived monthly patient demand	
1–10 requests	50 (70.4)
11–50 requests	6 (8.5)
51–100 requests	0 (0.0)
>100 requests	0 (0.0)
None	15 (21.1)
Incentives currently offered	
Yes	1 (1.4)
No	70 (98.6)
Perceived effectiveness of incentives	
Yes	23 (32.4)
Depending on the incentive type	9 (12.7)
No awareness should be the main driver	31 (43.7)
No—risk of misuse/abuse	8 (11.3)

Percentages may not sum to 100 due to rounding. ^†^ Totals exceed 100%, multiple responses and/or reporting inconsistencies; interpret cautiously.

## Data Availability

The data presented in this study are available on request from the corresponding author due to privacy considerations.

## Supplementary Materials

The following supporting information can be downloaded at: https://www.mdpi.com/article/10.3390/pharmacy14020061/s1, File S1: Questionnaire for Community Pharmacy Staff. File S2: Questionnaire for the General Public (Patients).

## Abbreviations

The following abbreviations are used in this manuscript:

AMR	Antimicrobial Resistance
EMA	European Medicines Agency
KAP	Knowledge, Attitudes, and Practices
MoH	Ministry of Health
OECD	Organisation for Economic Co-operation and Development
PiE	Pharmaceuticals in the Environment
WHO	World Health Organization
