# Supplementary material for: Knowledge, Attitudes, and Practices Regarding the Disposal of Unused and Expired Medicines in Romania During the Early Implementation of the 2023 Hospital-Based Collection Framework
Source: Pharmacy (Basel). 2026 Apr 16;14(2):61. doi: 10.3390/pharmacy14020061 (PMC13118957; doi:10.3390/pharmacy14020061)
Supplement: Supplementary file 1 [file pharmacy-14-00061-s001.zip › S1-Questionnaire for Community Pharmacy Staff.pdf]

Questionnaire for Community Pharmacy Staff

Title:
Practices and Attitudes of Community Pharmacy Staff Regarding the Collection of Unused and Expired Medicines

Instructions:
Please answer the following questions. The questionnaire is anonymous, and participation is voluntary.

Section 1. Professional and Demographic Data

Q1. Your role in the pharmacy:

- ☐ Pharmacist
- ☐ Assistant pharmacist
- ☐ Chief pharmacist
- ☐ Other: ___________

Q2. Pharmacy location:

- ☐ Urban
- ☐ Rural

Q3. Type of pharmacy:

- ☐ Independent pharmacy
- ☐ Chain pharmacy
- ☐ Hospital pharmacy

Q4. Age:

- ☐ 18–30
- ☐ 31–40
- ☐ 41–50
- ☐ >50

Q5. Sex:

- ☐ Male
- ☐ Female

Section 2. Current Practices

Q6. Does your pharmacy collect unused or expired medicines from the public?

- ☐ Yes, unused medicines only
- ☐ Yes, expired medicines only
- ☐ Yes, both
- ☐ No

Q7. How often are medicines collected in your pharmacy?

- ☐ Monthly
- ☐ Quarterly
- ☐ On demand
- ☐ Not applicable

Q8. What would be the preferred frequency for collecting such medicines?

- ☐ Monthly
- ☐ Quarterly
- ☐ On demand
- ☐ Not necessary

Section 3. Handling and System Organization

Q9. What happens to the collected medicines? *(⚠️ confirm if multi-select)*

- ☐ Destroyed according to regulations

- ☐ Transferred to a specialized waste management company
- ☐ Donated to hospitals
- ☐ Returned to stock

Section 4. Attitudes and Policy Perspectives

Q10. What is the most effective way to inform the public about medicine disposal?

- ☐ Informational leaflets
- ☐ Community campaigns
- ☐ Mass media (TV, radio, online)
- ☐ School-based education

Q11. Should medicine collection be a mandatory service in pharmacies?

- ☐ Yes
- ☐ No
- ☐ Depends on available resources
- ☐ No opinion

Section 5. Demand and Incentives

Q12. How many patients per month request to return unused or expired medicines?

- ☐ 1–10
- ☐ 11–50
- ☐ 51–100
- ☐ >100
- ☐ None

Q13. Does your pharmacy offer incentives to patients for returning medicines?

- ☐ Yes
- ☐ No

Q14. Do you think incentives are effective in increasing participation?

- ☐ Yes
- ☐ It depends
- ☐ No, awareness is more important
- ☐ No, due to risk of misuse
