# Supplementary material for: Knowledge, Attitudes, and Practices Regarding the Disposal of Unused and Expired Medicines in Romania During the Early Implementation of the 2023 Hospital-Based Collection Framework
Source: Pharmacy (Basel). 2026 Apr 16;14(2):61. doi: 10.3390/pharmacy14020061 (PMC13118957; doi:10.3390/pharmacy14020061)
Supplement: Supplementary file 1 [file pharmacy-14-00061-s001.zip › S2-Questionnaire for the General Public.pdf]

Questionnaire for the General Public (Patients)

Title:
Knowledge, Attitudes, and Practices Regarding the Disposal of Unused and Expired Medicines

Instructions:
Please answer the following questions. The questionnaire is anonymous, and participation is voluntary.

Section 1. Demographic Characteristics

Q1. Age:

- ☐ 18–30
- ☐ 31–40
- ☐ 41–50
- ☐ >50

Q2. Sex:

- ☐ Male
- ☐ Female

Q3. Place of residence:

- ☐ Urban
- ☐ Rural

Section 2. Practices

Q4. How do you usually dispose of unused or expired medicines? *(Multiple answers allowed)*

- ☐ Throw them in household trash
- ☐ Dispose of them in the sink/toilet
- ☐ Burn them
- ☐ Return them to a pharmacy
- ☐ Other: __________

Q5. How often do you dispose of medicines in inappropriate ways (e.g., trash, sink)?

- ☐ Very often
- ☐ Often
- ☐ Rarely
- ☐ Never

Q6. Have you ever tried to return unused or expired medicines to a pharmacy?

- ☐ Yes, and they were accepted
- ☐ Yes, but they were refused
- ☐ No, I have never tried

Section 3. Knowledge and Attitudes

Q7. Why do you think proper disposal of medicines is important? *(Multiple answers allowed)*

- ☐ To prevent accidental poisoning
- ☐ To protect the environment
- ☐ To prevent unauthorized access or misuse
- ☐ It is not important
- ☐ Other: ___________

Q8. How do you feel when you dispose of medicines improperly?

- ☐ Worried
- ☐ Guilty
- ☐ Uneasy
- ☐ Indifferent

Q9. Would you be willing to return unused or expired medicines if an appropriate system were available?

- ☐ Certainly yes
- ☐ Probably yes
- ☐ Not sure
- ☐ Certainly not

Section 4. Preferences and Incentives

Q10. What solutions would improve the collection of unused or expired medicines?

- ☐ Special collection containers/vending machines
- ☐ Legal obligation for medical units (pharmacies/hospitals)
- ☐ Public education and awareness campaigns
- ☐ No solution is needed

Q11. What would motivate you to return unused or expired medicines?

- ☐ Discounts or financial incentives
- ☐ Prize draws
- ☐ Protecting public health and the environment
- ☐ Nothing would motivate me
